# Clinical outcomes of modified left ventricular assist device driveline management

**DOI:** 10.1007/s10047-024-01482-8

**Published:** 2024-12-18

**Authors:** Shusuke Imaoka, Noriyuki Kashiyama, Daisuke Yoshioka, Shunsuke Saito, Takuji Kawamura, Ai Kawamura, Ryohei Matsuura, Yusuke Misumi, Koichi Toda, Shigeru Miyagawa

**Affiliations:** 1 Departments of Cardiovascular Surgery, Osaka University Graduate School of Medicine, 2-2, Yamadaoka, Suita Shi, Osaka, Osaka fu 565-0871 Japan; 2 Departments of Cardiovascular Surgery, Fukui Cardiovascular Hospital, Fukui, Japan; 3 Department of thoracic and cardiovascular surgery, Dokkyo Medical University Saitama Medical Center, Saitama, Japan

**Keywords:** Left ventricular assist device, Left ventricular assist device drive line, Drive line infection

## Abstract

Left ventricular assist devices (LVADs) are implanted in patients with heart failure to support cardiac circulation. However, no standardized methods have been established for LVAD driveline exit site management for the prevention of infections. Therefore, this study evaluated the efficacy of modified driveline management compared with that of conventional driveline management. We retrospectively assessed the outcomes of 262 patients who underwent continuous-flow LVAD implantation between January 2005 and March 2023 at Osaka University in Japan. In conventional driveline management, an LVAD driveline penetrates the skin along the body surface and is fixed near the penetration site (*n* = 224). In contrast, in our modified fixation method, the LVAD driveline vertically penetrates the skin to prevent ischemia at the driveline exit site and is fixed at a distant abdominal site to prevent the movement of the driveline exit site due to body movement (*n* = 38). The rates of freedom from LVAD driveline infection in patients with conventional driveline management were 86, 75, and 63% at 1, 2, and 3 years after LVAD implantation, respectively. The rate of freedom from LVAD driveline infection in patients managed by the modified fixation method was 91% at 1, 2, as well as 3 years after LVAD implantation. The freedom rates from LVAD driveline infection in the patients with modified fixation method was lower than in the patients with the conventional method (*p* = 0.04). Our study revealed that the modified fixation method may offer the possibility for preventing LVAD driveline infection.

## Introduction

The clinical outcomes of durable left ventricular assist device (LVAD) implantation have improved with device improvements; however, it is reported that 15% to 30% of patients undergo this procedure experience LVAD driveline infection, and the rate of freedom from LVAD driveline infection has not improved significantly [1–6]. LVAD driveline exit site trauma can cause LVAD driveline infection, followed by the spread of the infection to the pump components. Once infection spreads to the pump components, the prognosis is poor without the use of effective invasive treatment strategies [7–9]. Trauma at the LVAD driveline exit site, induced by LVAD driveline movement, is one of the causes of LVAD driveline infection [10]. Thus, careful management of the LVAD driveline exit site is crucial for preventing LVAD driveline infections [8]. Some studies have reported methods of driveline management that immobilize the LVAD driveline exit site [11–13]. However, no standardized methods have been established for the management of the LVAD driveline exit site to prevent driveline infection [8]. Preventing LVAD driveline infection, LVAD driveline exit site should be immobilized for preventing ischemia and trauma at driveline exit site. Therefore, we have begun to apply the modified driveline management. In the modified driveline management, LVAD driveline was penetrated vertically into the skin for preventing skin ischemia due to oppression of LVAD driveline itself and fixed at a distant site on the front of the abdomen for preventing movement at LVAD driveline exit site by body movement. In this study, we considered the modified driveline management could prevent LVAD driveline infection more than the conventional driveline management and compared the rates of freedom from LVAD driveline infection between the patients with conventional driveline management and modified driveline management.

## Materials and methods

### Patients

We enrolled 262 patients who underwent their first continuous-flow LVAD implantation for end-stage heart failure, including bridge-to-bridge, bridge-to-transplantation, and destination therapy, between January 2005 and March 2023. Patients who underwent conversion from one implantable LVAD to another were excluded. Among these patients, 224 patients were performed the conventional driveline management and 38 patients were performed the modified fixation method. We evaluated the effectiveness of the modified driveline management in comparison with the conventional driveline management. The mean follow-up duration was 854 ± 648 days. All follow-up examinations were completed on July 31, 2023.

All patients and their families provided informed consent to participate in related clinical studies before LVAD implantation.

### Conventional and modified driveline management

At our hospital, LVAD driveline was positioned between rectus abdominal muscle and anterior layer of rectus from end of thoracic cavity to just below skin penetration site at LVAD implantation. We conduct VAD rounds to evaluate and provide advice on driveline management. These rounds are usually conducted once a week by a team of experts, including cardiovascular surgeons, VAD coordinators, and wound-care nurses. We used 0.5% chlorhexidine gluconate or saline for daily care at the LVAD driveline exit site. No special dressings were used, and the LVAD driveline exit site was protected with clean gauze. During the patients’ stay in the intensive care unit (ICU) after LVAD implantation, the cardiovascular surgeon was responsible for the daily care of the LVAD driveline exit site. After leaving the ICU, the wound-care nurses took over this responsibility. Patients received instructions on driveline care from the wound-care nurses until they or their caregivers demonstrated satisfactory cleaning skills. Patient care skills were also assessed during the VAD rounds. After discharge from the hospital, the patients or their caregivers were responsible for driveline care. When the patients took showers, the LVAD driveline exit site remained waterproof. The status of the LVAD driveline exit site was checked during the monthly outpatient treatment by VAD-expert medical doctors and the VAD coordinator nurses.

**Fig. 1:**
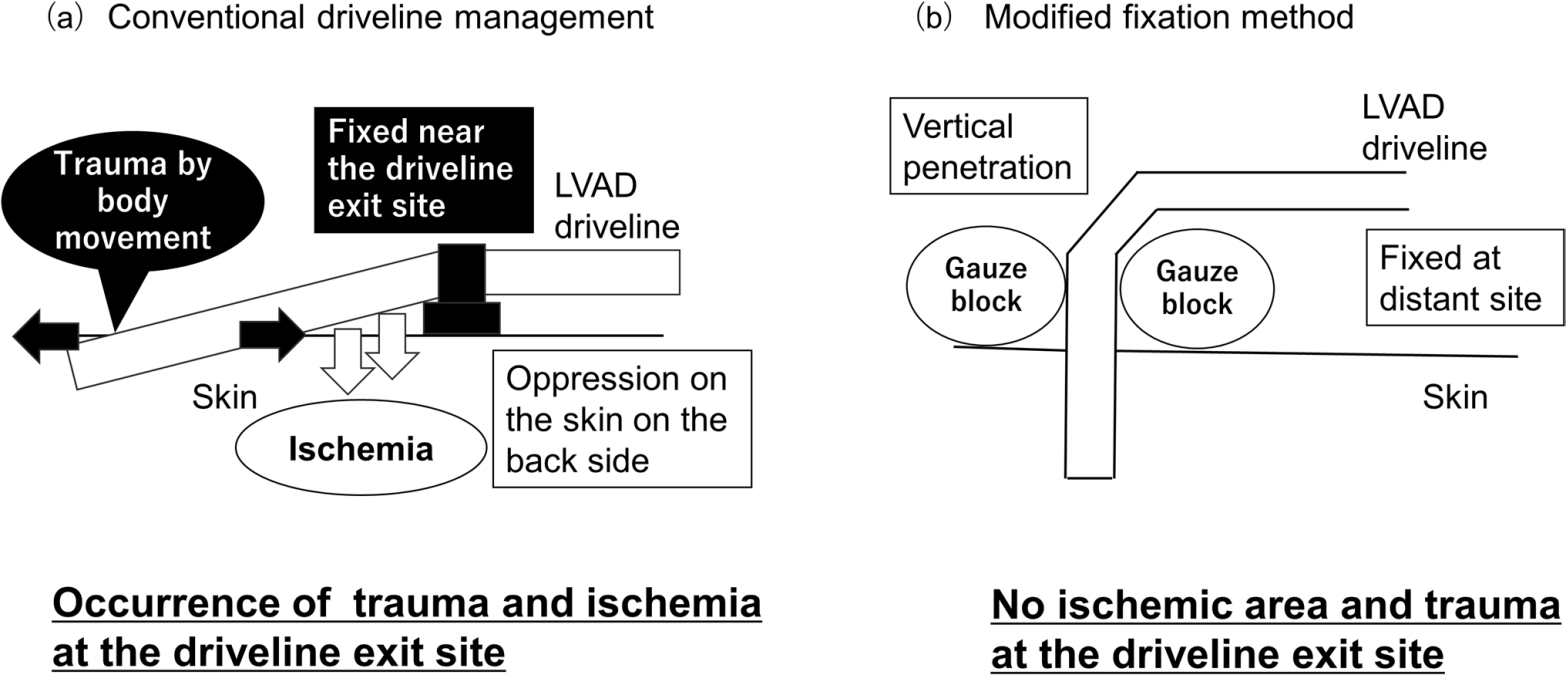
Comparison of concepts in conventional driveline management and modified fixation method. **a** Concept and problems of conventional driveline management. **b** Concept of the modified fixation method. Abbreviations: *LVAD* left ventricular assist device

**Fig. 2:**
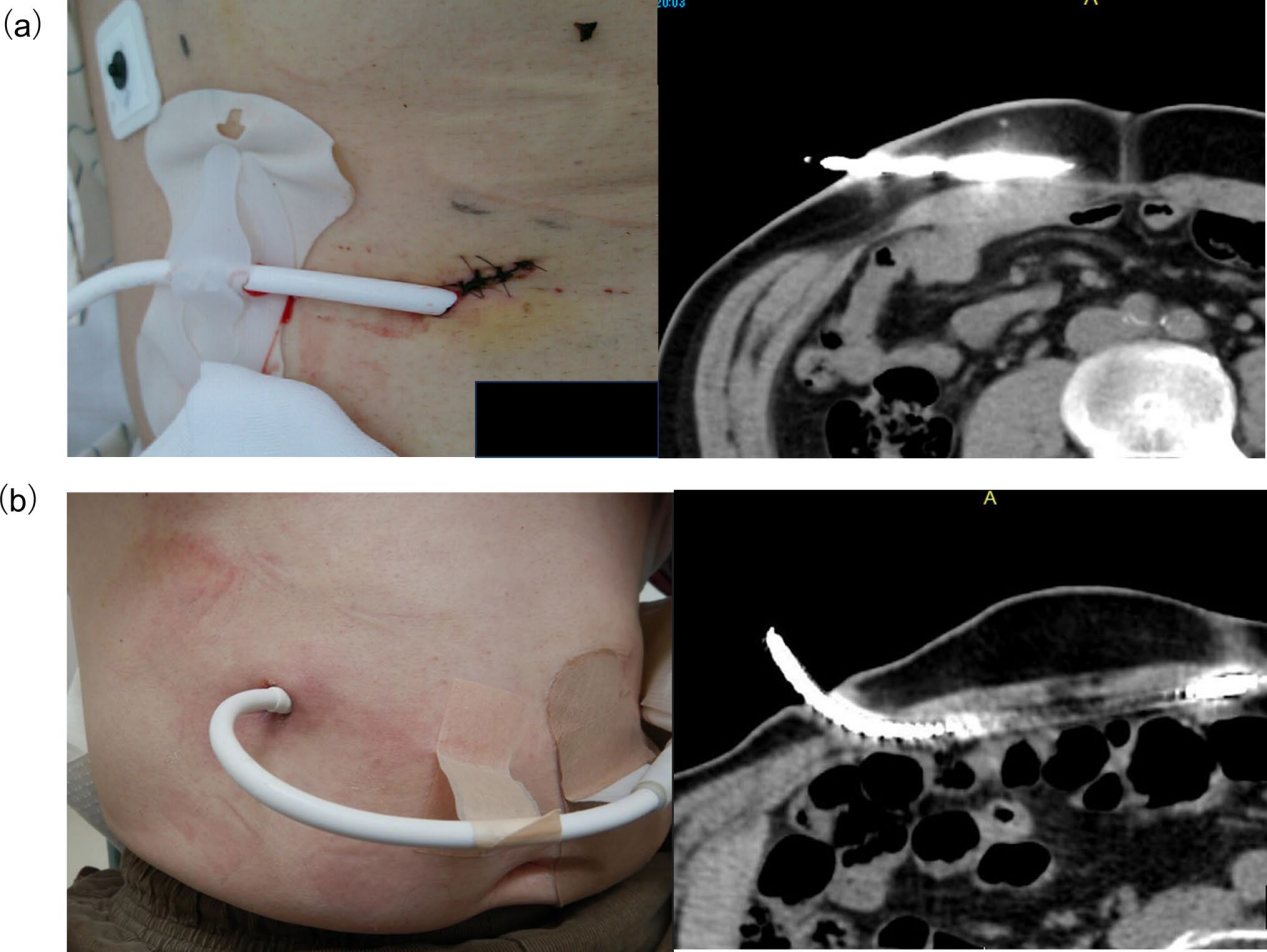
Comparison of conventional driveline management and modified fixation method. **a** Picture and computed tomography images of skin penetration of the LVAD driveline in a patient with conventional driveline management. **b** Picture and computed tomography images of skin penetration of the LVAD driveline in a patient with the modified fixation method

In conventional driveline management (Figs. 1a, 2a), the LVAD driveline penetrated the skin along the body surface and was fixed near the penetration site. This approach led to oppression on the skin on the back side of the LVAD driveline at the exit site, potentially causing skin tissue ischemia, resulting in trauma and ulceration at the LVAD driveline exit site. The movement of the LVAD driveline exit site due to body movements also increased the risk of trauma and driveline infection. Discharge from the LVAD driveline exit site was frequently monitored. Therefore, the fixed position and penetration angle of the LVAD driveline from the skin were changed during the VAD rounds.

The modified fixation method (Figs. 1b, 2b) was implemented to manage the problems associated with conventional driveline management. This modified fixation method involves vertical penetration of the LVAD driveline into the skin. Subsequently, the LVAD driveline was fixed at a distant site on the front of the abdomen. A gauze block was used to assist in vertically fixing the LVAD driveline to the skin. Problems concerning the skin on the back of the LVAD driveline at the exit site were avoided by penetrating the LVAD driveline vertically from the skin. The movement of the LVAD driveline exit site was suppressed by fixing the driveline at a distance from the LVAD driveline exit site (on the front of the abdomen), relieving the stress caused by the movement of the body to the LVAD driveline exit site. The fixed LVAD driveline was protected with gauze, and a special abdominal band was placed over the area to protect the entire LVAD driveline. Overall, the modified fixation method prevented both the ischemia of the skin on the back side of the LVAD driveline and the trauma at the LVAD driveline exit site.

### Definition of LVAD driveline infections

In this study, an LVAD driveline infection was defined as a deep driveline infection that required treatments including surgical intervention and intravenous administration of antibiotics. Deep driveline infection involved infectious symptoms, including a local increase in temperature and pain around the exit site, elevated inflammatory reaction level, and positive wound culture and abscess from the deep soft tissue around the LVAD driveline observed during surgical debridement for the LVAD driveline infection [7, 14].

### Data collection

Patient data included baseline characteristics, etiology, comorbidities, preoperative hemodynamics, laboratory values, echocardiographic parameters, LVAD type, method of driveline management, and duration of LVAD implantation. All patient data were collected from the electronic medical and operative records.

### Statistical analyses

Continuous variables were presented as the median (interquartile range) or mean ± standard deviation. All statistical analyses were performed using JMP 16.0 (SAS Inc., Cary, NC, USA). Categorical variables were summarized as frequencies and percentages and compared among groups using Chi-square or Fisher’s exact tests. All *p*-values for the statistical analyses were two-tailed, and statistical significance was set at *p* < 0.05. Kaplan–Meier analysis was used to calculate the rates of freedom from LVAD driveline infections. Propensity score matching (1:2 matching, with the nearest neighbor matching without replacement) was performed to adjust for significant differences in risk factors of LVAD driveline infection (age, body mass index (BMI), preoperative albumin and preoperative incidence of diabetes mellitus (DM)) between patients with conventional driveline management and modified fixation method.

## Results

### Patient characteristics

The preoperative characteristics of the 262 enrolled patients are presented in Table 1. The mean age of the patients was 47 (35–56) years, and 181 (69%) were men. LVAD driveline infection occurred in 72 patients during follow-up. The rates of freedom from LVAD driveline infection at 1, 3, and 5 years were 87, 66, and 58%, respectively.

**Table 1:** Comparison of patient background between the patients with conventional driveline management and modified fixation method

	All	Conventional management	Modified fixation method	*p*-value
	*n* = 262	*n* = 224	*n* = 38	
Age at LVAD implantation(y)	47 (35–56)	46 (33–55)	53 (44–57)	< 0.01*
Female, *n* (%)	81 (31)	73 (33)	8 (21)	0.14
Body surface area (m^2^)	1.6 (1.5–1.7)	1.6 (1.5–1.7)	1.7 (1.5–1.7)	0.07
Body mass index (kg/m^2^)	20 (18–22)	20 (18–22)	21 (19–24)	0.06
INTERMACS profile				
Profile I, *n* (%)	28 (11)	25 (11)	3 (8)	0.91
Profile II, *n* (%)	81 (31)	75 (33)	6 (16)	0.03*
Profile III, *n* (%)	116 (44)	94 (42)	22 (58)	0.11
Profile IV, *n* (%)	17 (6)	11 (5)	6 (16)	0.02*
Bridge-to-Bridge, *n* (%)	20 (7)	19 (8)	1 (3)	0.16
Etiology				
Ischemic cardiomyopathy, *n* (%)	38 (15)	30 (13)	8 (21)	0.26
Idiopathic dilated cardiomyopathy, *n* (%)	138 (53)	119 (53)	19 (50)	0.62
Hypertrophic cardio myopathy, *n* (%)	37 (14)	32 (14)	5 (13)	0.81
Device				
HeartMate II, *n* (%)	79 (30)	79 (35)	0 (0)	< 0.01*
HeartMate 3, *n* (%)	43 (16)	11 (5)	32 (84)	< 0.01*
Others, *n* (%)	140 (53)	134 (60)	6 (16)	< 0.01*
Preoperative hemodynamics				
Intropes, *n* (%)	237 (90)	201 (90)	36 (95)	0.30
Intra-aortic balloon pumping, *n* (%)	57 (22)	53 (24)	4 (11)	0.05
Intubation, *n* (%)	38 (15)	35 (16)	3 (8)	0.18
VA-ECMO or percutaneous LVAD, *n* (%)	25 (10)	19 (8)	6 (16)	0.19
Preoperative echocardiographic parameters				
LVDd (mm)	69 ± 13	69 ± 13	72 ± 12	0.17
LVDs (mm)	63 ± 14	63 ± 14	66 ± 14	0.18
LVEF (%)	21 ± 9	21 ± 9	20 ± 9	0.60
Preoperative laboratory valuables				
Hemoglobin (g/dL)	11.6 (10.2–13.1)	11.4 (10.2–12.9)	12.3 (11.1–13.9)	0.90
WBC (× 1000/mm^3^)	6.4 (4.8–7.9)	6.4 (4.8–8.0)	6.1 (4.7–6.7)	0.56
CRP (mg/dL)	0.3 (0.1–1.7)	0.4 (0.1–1.8)	0.2 (0.0–0.5)	0.40
BUN (mg/dL)	18 (13–24)	18 (13–24)	18 (13–22)	0.58
Creatinine (mg/dL)	1.0 (0.7–1.2)	1.0 (0.7–1.2)	1.0 (0.8–1.2)	0.70
AST (IU/dL)	26 (20–36)	26 (21–36)	24 (18–34)	0.55
ALT (IU/dL)	21 (15–36)	22 (15–36)	21 (15–35)	0.40
T-bil (mg/dL)	0.9 (0.6–1.3)	0.9 (0.6–1.4)	0.8 (0.5–1.1)	0.32
Albumin (mg/dL)	3.8 (3.3–4.2)	3.8 (3.2–4.2)	3.9 (3.5–4.2)	0.13
Others				
DM, *n* (%)	49 (19)	15 (21)	9 (24)	0.45
Emergency surgery, *n* (%)	83 (32)	73 (33)	10 (26)	0.43

*LVAD* left ventricle assist device, *ICM* ischemic cardiomyopathy, *DCM* idiopathic dilated cardiomyopathy, *HCM* hypertrophic cardio myopathy, *IABP* intra-aortic balloon pumping, *VA-ECMO* veno-arterial extracorporeal membrane, *LVDd* left ventricular internal dimension in diastole, *LVDs* left ventricular internal dimension in systole, *LVEF* left ventricular ejection fraction, *WBC* white blood cell count, *CRP* C-reactive protein, *BUN* blood urea nitrogen, *AST* aspartate transaminase, *ALT* alanine transaminase, *T-bil* total bilirubin, *DM* diabetes mellitus

### Comparison of patient background between the patients with conventional driveline management and modified fixation method

The comparison of patient background between the patients with conventional driveline management and modified fixation method are presented in Table 1. No significant difference was observed in gender, body surface area, BMI, etiology, preoperative echocardiographic parameters, preoperative, laboratory valuables and preoperative incidence of DM. The patients with modified fixation method were older than the patients with conventional driveline management (46 (33–55) vs. 53 (44–57) years old, *p* < 0.01). Regarding of INTERMACS profile, there are more patients of Profile II in the patients with conventional driveline management and more patients of Profile IV in the patients with modified fixation method (Profile II: 75[33%] vs 6[16%], *p* = 0.03, Profile IV: 11[5%] vs 6[16%], *p* = 0.02). The device type in patients with modified fixation method was almost HeartMate 3 and the device type in patients with conventional driveline management was almost HeartMate II and other devices (HeartMate II: 79[35%] vs 0[0%], *p* < 0.01, HeartMate 3: 11[5%] vs 32[84%], *p* < 0.01, other devices: 134[60%] vs 6[16%]).

### Comparison of the rate of freedom from LVAD driveline infection between the patients with conventional driveline management and the modified fixation method

**Fig. 3:**
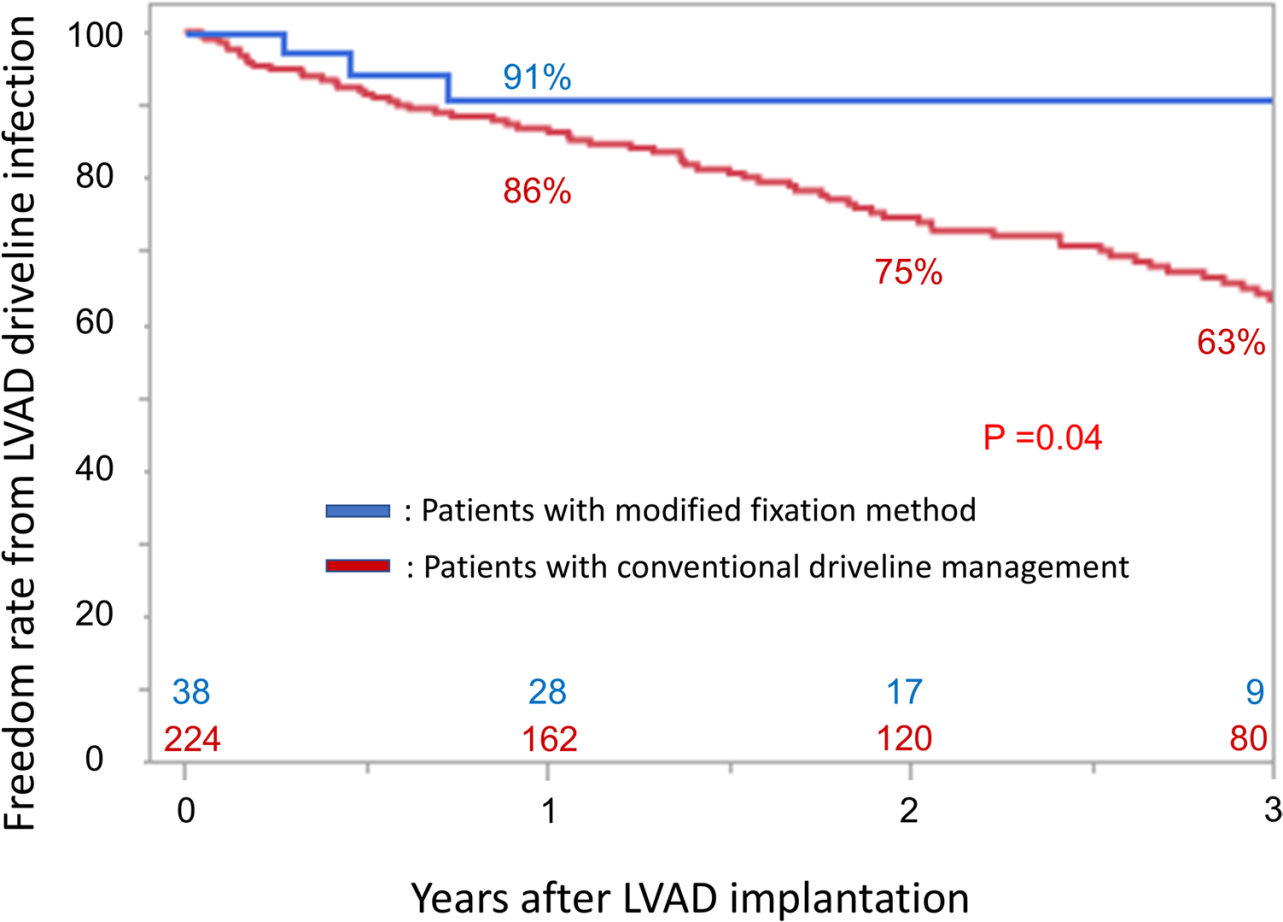
Freedom rate from left ventricular assist device driveline infection in patients with conventional driveline management and the modified fixation method. Abbreviations: *LVAD* left ventricular assist device

The rates of freedom from LVAD driveline infection in patients with conventional driveline management were 86, 75, and 63% at 1, 2, and 3 years after LVAD implantation, respectively. Meanwhile, the rate of freedom from LVAD driveline infection in patients managed with the modified fixation method was 91% at 1, 2, as well as 3 years after LVAD implantation. The modified fixation method significantly reduced the occurrence of LVAD driveline infection, compared with conventional driveline management (*p* = 0.04; Fig. 3).

### Comparison of the rate of freedom from LVAD driveline infection between the patients with conventional driveline management and the modified fixation method after the propensity score matching

**Fig. 4:**
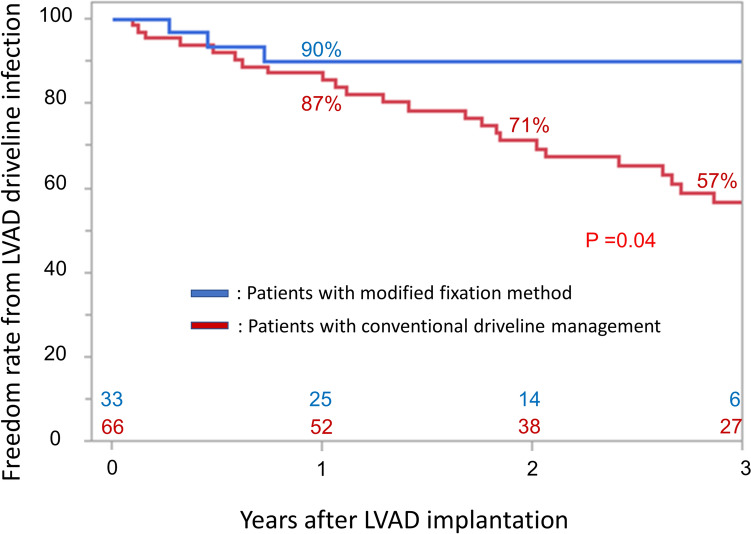
Freedom rate from left ventricular assist device driveline infection in patients with conventional driveline management and the modified fixation method after the propensity score matching. Abbreviations: *LVAD* left ventricular assist device

It was observed no significant difference in the patient background between the patients with conventional driveline management and the modified fixation method after the propensity score matching regarding age, BMI, preoperative albumin and preoperative incidence of DM (Table 2). The rates of freedom from LVAD driveline infection in patients with conventional driveline management after the propensity score matching were 87, 71, and 57% at 1, 2, and 3 years after LVAD implantation, respectively. Meanwhile, the rate of freedom from LVAD driveline infection in patients managed with the modified fixation method after the propensity score matching was 90% at 1, 2, as well as 3 years after LVAD implantation. The modified fixation method significantly reduced the occurrence of LVAD driveline infection, compared with conventional driveline management (*p* = 0.04; Fig. 4).

**Table 2:** Comparison of patient background after the propensity score matching

	Conventional management	Modified fixation method	*p*-value
	*n* = 66	*n* = 33	
Age at LVAD implantation(y)	52 (42–59)	53 (42–57)	0.89
Female, *n* (%)	19 (29)	7 (21)	0.39
Body surface area (m^2^)	1.6 (1.6–1.8)	1.7 (1.5–1.7)	0.99
Body mass index (kg/m^2^)	21 (18–23)	20 (18–22)	0.63
INTERMACS profile			
Profile I, *n* (%)	3 (5)	2 (6)	0.69
Profile II, *n* (%)	20 (30)	5 (15)	0.13
Profile III, *n* (%)	36 (55)	21 (64)	0.53
Profile IV, *n* (%)	2 (3)	4 (12)	0.07
Bridge-to-Bridge, *n* (%)	5 (8)	1 (3)	0.38
Etiology			
Ischemic cardiomyopathy, *n* (%)	13 (20)	7 (21)	0.88
Idiopathic dilated cardiomyopathy, *n* (%)	32 (48)	15 (45)	0.72
Hypertrophic cardio myopathy, *n* (%)	11 (17)	5 (15)	0.82
Device			
HeartMate II, *n* (%)	35 (53)	0 (0)	< 0.01*
HeartMate 3, *n* (%)	5 (8)	30 (91)	< 0.01*
HeartWare, *n* (%)	7 (11)	3 (9)	0.81
Others, *n* (%)	19 (29)	0 (0)	< 0.01*
Preoperative hemodynamics			
Intropes, *n* (%)	59 (89)	32 (97)	0.16
Intra-aortic balloon pumping, *n* (%)	13 (20)	3 (9)	0.16
Intubation, *n* (%)	3 (5)	3 (9)	0.38
VA-ECMO or percutaneous LVAD, *n* (%)	4 (6)	5 (15)	0.16
Preoperative echocardiographic parameters			
LVDd (mm)	71 ± 14	71 ± 12	0.98
LVDs (mm)	66 ± 14	66 ± 14	0.95
LVEF (%)	20 ± 9	20 ± 10	0.99
Preoperative laboratory valuables			
Hemoglobin (g/dL)	11.8 (10.3–13.5)	12.2 (10.2–13.9)	0.27
WBC (× 1000/mm^3^)	5.8 (4.5–7.7)	6.1 (4.6–6.7)	0.73
CRP (mg/dL)	0.2 (0.0–1.2)	0.2 (0.0–0.6)	0.48
BUN (mg/dL)	18 (14–23)	18 (14–22)	0.78
Creatinine (mg/dL)	1.0 (0.9–1.3)	1.0 (0.8–1.2)	0.32
AST (IU/dL)	24 (20–33)	23 (18–32)	0.21
ALT (IU/dL)	18 (14–29)	21 (15–37)	0.23
T-bil (mg/dL)	0.9 (0.7–1.2)	0.9 (0.5–1.1)	0.61
Albumin (mg/dL)	3.9 (3.6–4.1)	3.9 (3.5–4.2)	0.81
Others			
DM, *n* (%)	16 (24)	9 (27)	0.74
Emergency surgery, *n* (%)	12 (18)	9 (27)	0.30

*LVAD* left ventricle assist device, *ICM* ischemic cardiomyopathy, *DCM* idiopathic dilated cardiomyopathy, *HCM* hypertrophic cardio myopathy, *IABP* intra-aortic balloon pumping, *VA-ECMO* veno-arterial extracorporeal membrane, *LVDd* left ventricular internal dimension in diastole, *LVDs* left ventricular internal dimension in systole, *LVEF* left ventricular ejection fraction, *WBC* white blood cell count, *CRP* C-reactive protein, *BUN* blood urea nitrogen, *AST* aspartate transaminase, *ALT* alanine transaminase, *T-bil* total bilirubin, *DM* diabetes mellitus

### The clinical course of cases with LVAD driveline infection after the application of the modified fixation method

LVAD driveline infection was observed in three patients after the application of the modified fixation method of LVAD driveline. The pathogenic bacteria were methicillin-susceptible *Staphylococcus aureus* in two cases and methicillin-resistant* S. aureus* in one case. Drainage therapy, including wound debridement, negative-pressure wound therapy, and antibiotic treatment, was performed in all cases. In two cases, the recurrence was not observed after the treatment for LVAD drive line infection. In these cases, the infection spread from the LVAD driveline exit site, and the wound was debrided from the LVAD driveline exit site. After the wound culture turned negative, the LVAD driveline was repositioned at the new LVAD driveline exit site and fixed using the modified fixation method. Cultures from the new driveline exit site and debrided wound remained negative, and the wound closed. The two patients had no recurrence of LVAD driveline infection for 484 and 677 days, respectively. In the other case, the recurrence of LVAD driveline infection was occurred after the treatment for LVAD drive line infection. In this case, the infection spread to the subcutaneous LVAD driveline around the umbilicus, and the wound was debrided. The wound culture after debridement remained positive, and negative pressure wound therapy and antibiotic treatment were continued after hospital discharge. The LVAD driveline infection recurred 2 months after discharge from the hospital. Pus was observed in the wound around the umbilicus. Subsequently, the wound was debrided again. However, the infection spread to the LVAD driveline near the pump body, and an LVAD pump exchange was finally performed.

## Discussion

The modified fixation method tended to prevent LVAD driveline infection, compared with conventional driveline management. Some previous studies have reported the use of management kits for immobilizing the LVAD driveline exit site, preventing LVAD driveline infection [11–13]. The modified fixation method is considered one of the most effective methods for preventing LVAD driveline infection by fixing the LVAD driveline exit site. Furthermore, unlike conventional methods, the modified fixation method of the LVAD driveline does not lead to skin tissue ischemia at the LVAD driveline exit site.

The modified fixation method of LVAD driveline was applied for the patients in the last 4 years and the conventional driveline management was applied for the patients from the 15 years to 5 years ago. During this time, percutaneous LVAD (Impella) and new type LVAD (HeartMate 3) was developed and destination therapy was initiated. These factors may influence the difference of patient background between the patients with conventional driveline management and modified fixation method. Previous studies have reported the risk factors for LVAD driveline infection, including younger age, higher BMI, and DM comorbidity, suggesting that higher level of physical activity in the younger population and decreased immune function in patients with higher BMI and comorbid DM increase the risk of driveline exit-site trauma [1–4]. In this study, the BMI and comorbid DM were not included in the difference of patient background and the age of patients was included in the difference of patient background. The age of patients may influence the result in this study. Some previous studies and Japanese registry for Mechanically Assisted Circulatory Support in 2024 was reported that the freedom rate from LVAD driveline infection was about 80%, 70% and 65% at 1, 2 and 3 years after LVAD implantation, respectively [1, 2, 5, 10]. However, the freedom rate from LVAD driveline infection in the patients with modified fixation method was 91% at 1, 2, as well as 3 years after LVAD implantation and the modified fixation method may reduce the occurrence of LVAD driveline infection.

In patients with LVAD driveline infections treated by the modified fixation method of the driveline, poor compliance of patients or healthcare providers regarding driveline movement and attention to wound care was observed. In this study, LVAD driveline infections were caused by *S. aureus* in all cases. *S. aureus* is reportedly the predominant bacteria in LVAD-associated infections, including LVAD driveline infections [5], and shows a strong virulence in LVAD driveline infections [15]. Even with the modified fixation method, once trauma is induced at the LVAD driveline exit site,* S. aureus* infections may occur at the LVAD driveline. The wound care and fixing method of driveline required for the modified fixation method is complex. Therefore, patient education regarding would care is crucial. Appropriate driveline management and good compliance with healthcare providers’ instructions are essential for preventing LVAD driveline infections.

### Limitations

In this study, the modified fixation method of LVAD driveline was applied for patients in the last 4 years and mainly in patients who underwent implantation of the HeartMate 3 device. However, conventional driveline management was applied for patients who underwent implantation of a variety of devices, which included HeartWare, DuraHeart, EVAHEART, EVAHEART II, Jarvik2000, HeartMate II, and HeartMate 3. Therefore, the possibility of the material of the LVAD driveline having influenced the results cannot be ruled out.

## Conclusions

The modified fixation method of the LVAD driveline may offer the possibility of preventing LVAD driveline infection.

## Data Availability

The deidentified participant data will not be shared.
